# Traumatic brain injury among people who are homeless: a systematic review

**DOI:** 10.1186/1471-2458-12-1059

**Published:** 2012-12-08

**Authors:** Jane Topolovec-Vranic, Naomi Ennis, Angela Colantonio, Michael D Cusimano, Stephen W Hwang, Pia Kontos, Donna Ouchterlony, Vicky Stergiopoulos

**Affiliations:** 1 Trauma and Neurosurgery Program, Keenan Research Center of the Li Ka Shing Knowledge Institute, St. Michael’s Hospital; Department of Occupational Science and Occupational Therapy, University of Toronto, 30 Bond Street, Bond 3-012, Toronto, ON M5B 1W8, Canada; 2 Head Injury Clinic, Trauma and Neurosurgery Program, St. Michael's Hospital, Toronto, Canada; 3 Toronto Rehabilitation Institute; Occupational Science and Occupational Therapy, Rehabilitation and Public Health Sciences, University of Toronto, Toronto, Canada; 4 Injury Prevention Research Office, Keenan Research Center of the Li Ka Shing Knowledge Institute, St. Michael’s Hospital; Division of Neurosurgery, University of Toronto, Toronto, Canada; 5 Center for Research on Inner City Health, Keenan Research Center of the Li Ka Shing Knowledge Institute, St. Michael’s Hospital; Division of General Internal Medicine, Department of Medicine, University of Toronto, Toronto, Canada; 6 Toronto Rehabilitation Institute-University Health Network; Dalla Lana School of Public Health, University of Toronto, Toronto, Canada; 7 Head Injury Clinic, Trauma and Neurosurgery Program, St. Michael's Hospital, Toronto, Canada; 8 Center for Research on Inner City Health, Keenan Research Center of the Li Ka Shing Knowledge Institute, St. Michael’s Hospital; Department of Psychiatry, University of Toronto, Toronto, Canada

**Keywords:** Traumatic brain injury, Homelessness, Systematic review

## Abstract

**Background:**

Homelessness and poverty are important social problems, and reducing the prevalence of homelessness and the incidence of injury and illness among people who are homeless would have significant financial, societal, and individual implications. Recent research has identified high rates of traumatic brain injury (TBI) among this population, but to date there has not been a review of the literature on this topic. The objective of this systematic review was to review the current state of the literature on TBI and homelessness in order to identify knowledge gaps and direct future research.

**Methods:**

A systematic literature search was conducted in PsycINFO (1887–2012), Embase (1947–2012), and MEDLINE/Pubmed (1966–2012) to identify all published research studies on TBI and homelessness. Data on setting, sampling, outcome measures, and rate of TBI were extracted from these studies.

**Results:**

Eight research studies were identified. The rate of TBI among samples of persons who were homeless varied across studies, ranging from 8%-53%. Across the studies there was generally little information to adequately describe the research setting, sample sizes were small and consisted mainly of adult males, demographic information was not well described, and validated screening tools were rarely used. The methodological quality of the studies included was generally moderate and there was little information to illustrate that the studies were adequately powered or that study samples were representative of the source population. There was also an absence of qualitative studies in the literature.

**Conclusions:**

The rate of TBI is higher among persons who are homeless as compared to the general population. Both descriptive and interventional studies of individuals who are homeless should include a psychometrically sound measure of history of TBI and related disability. Education of caregivers of persons who are at risk of becoming, or are homeless, should involve training on TBI. Dissemination of knowledge to key stakeholders such as people who are homeless, their families, and public policy makers is also advocated.

## Background

Homelessness and poverty are among the most critical social problems in Canada today, as reported by Canadians recently polled for the Salvation Army’s *Poverty Shouldn’t be a Life Sentence* report [1]. The number of individuals affected by homelessness in Canada is substantial: approximately one in nine (12%) of the sample of 1,000 adult respondents of the poll indicated that they had experienced homelessness or came close to experiencing homelessness in their life [1]. In 2008, the Toronto, Canada shelter system was used by 27,256 adults; the projected 2010 operating budget for these shelters was $48,473,622 [2].

In addition to the societal and financial burdens of homelessness, individuals who are homeless often suffer from serious health conditions and are at increased risk of death [3-5]. In the larger context of the healthcare system, homeless persons use the most expensive interventions (e.g. emergency rooms, inpatient units), and on average, spend four more days in hospital each year than non-homeless people [6]. Thus, the potential to reduce the rate of homelessness and the incidence of injury and illness among people who are homeless has significant financial, societal, and individual implications.

Recently researchers have begun to explore the inter-relationship between homelessness and traumatic brain injury (TBI) [7]. TBI is defined as “a blow or jolt to the head or a penetrating head injury that disrupts the function of the brain” [8,9]. The severity of a TBI may range from mild, characterized by a brief change in mental status, to severe, characterized by an extended period of unconsciousness or amnesia after the injury [9]. TBI often occurs among young persons, affecting prime working years [9,10]. Evidence, predominantly from individuals with more severe TBI, suggests that cognitive, physical and emotional consequences may persist and place individuals at risk for social failure. TBI is associated with low subsequent employment rates which can contribute to a downward spiral to homelessness [11]. It is also suggested that in the homeless population, cognitive impairment may increase the risk of remaining homeless [12], illustrating the potential for TBIs to contribute to the chronicity of homelessness.

Certain health conditions are common to both the homeless and TBI populations. For instance, the rate of mental disorders among individuals who are homeless is high and is estimated to be between 80%-90% in countries such as the US and Canada [13]. Similarly, psychiatric disorders are also highly prevalent in individuals post-TBI, with estimates of rates ranging between 18%-65% [14-16]. However, as Silver and Felix (1999) point out, “Unfortunately the psychiatric impairments caused by TBI often go unrecognized” in homeless populations [17]. Cognitive impairment, a common sequelae of TBI, is also commonly observed in individuals who are homeless [18] with recent studies demonstrating impairment among 80% of homeless persons. However, the potential relationship between past TBI and the cognitive impairments observed amongst individuals who are homeless is unclear.

A greater understanding of the link between homelessness and TBI, including more accurate measurements of rates and impairments associated with TBI, is necessary in order to tailor prevention and intervention programs aimed at reducing the incidence and managing the symptoms of TBI among people who are homeless or at risk of becoming homeless. The objectives of this systematic review were twofold: 1) to summarize the current published literature related to TBI among people who are homeless and 2) to determine the gaps in the literature in order to direct future research. The findings of this review could be used to guide both future research and clinical care to reduce suffering and improve outcomes among vulnerable populations.

## Methods

A systematic literature review was conducted following PRISMA (Preferred Reporting Items for Systematic Reviews and Meta-analyses) [19] suggestions. A protocol for this study has not previously been published.

### Eligibility criteria

All identified studies were screened and reviewed for relevance by two authors (JTV and NE). The selection criteria were broad and inclusive in order to capture all relevant research on the topic. All original research published in peer-reviewed journals were included for review. Only the following were excluded: 1) studies that did not have samples exclusively comprised of homeless people; 2) studies solely examining non-TBI; 3) original research studies that have not been published in peer-reviewed journals; and 4) review articles.

### Information sources

A systematic literature search was conducted in PsycINFO (1887–2012), Embase (1947–2012), and MEDLINE/Pubmed (1966–2012) using the search terms: (“Traumatic Brain Injury” OR “Head Injury” OR “Brain Injury” OR “Head Trauma” OR “Brain Damage”) AND (“Homeless” OR “Homelessness” OR “Rooflessness”). Manual searches were performed using reference lists from relevant papers. The search concluded on October 1, 2012.

### Data extraction

Titles and abstracts of research studies were screened to identify studies of likely relevance and full papers were subsequently obtained. Methodological data on sampling, outcome measures and the rates of TBI among the study samples were extracted from all selected research studies.

### Methodological quality of studies

The Downs and Black checklist (1998) was used to evaluate the overall methodological quality of the included studies. This checklist measures quality of both randomized clinical trials and nonrandomized studies using several items distributed across the following subscales: reporting, external validity, internal validity (bias and confounding), and power [20]. The checklist demonstrates good test-retest reliability (r=.88), inter-rater reliability (r=.75), and internal consistency (Kruder-Richardson formula 20=.89) [20]. The original checklist contains 27 items however ten items relating to interventional trials (e.g. reporting of treatment groups, attempts made to blind subjects and those measuring outcomes, internal validity (selection bias) of treatment and control groups) were excluded from this review as no interventional studies were identified. An article could achieve a total possible score of 17 and the higher the score, the better the methodological quality of the study. Specifically, quality ratings between 12 and 17 were considered good, six to 12 moderate and less than six poor. Two raters (NE, J.R) applied this checklist to all studies. Inter-rater reliability ranged from 85%-100% on each article reviewed. The raters met to discuss any discrepancies in the scores of each checklist item and consensus was achieved for each item discrepancy.

## Results

### Study selection

**Figure 1:**
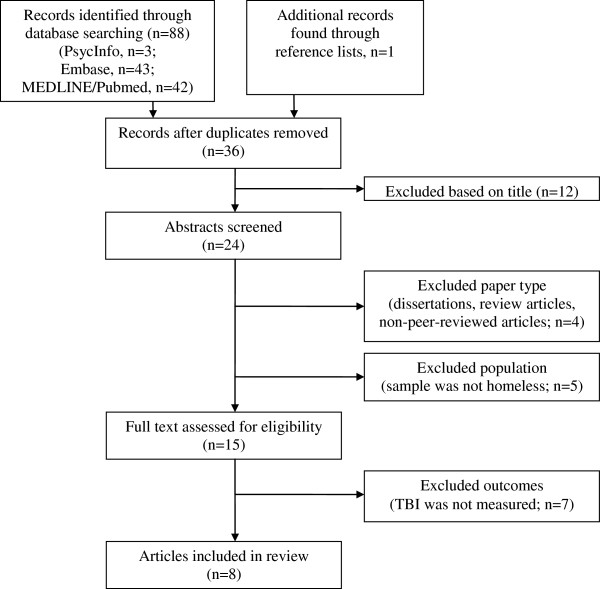
Systematic search strategy.

The search yielded 89 results (Figure 1). Of these, 53 were duplicates and twelve were excluded based on their titles, leaving 24 abstracts that were further screened for relevance. From these 24 abstracts, four papers were excluded because they were either dissertations (n=2), review articles (n=1), or non-peer reviewed research articles (n=1) and five papers were excluded because the samples were not exclusively comprised of people who were homeless. Seven studies were further excluded because TBI was not measured, leaving eight studies that were included for review (Table 1). The studies were published between 1996–2012.

**Table 1:** Study characteristics (N=8)

**Research study**	**Quality**^**1**^** rating**	**Setting**	**Sample size (% male); mean age in years (SD); mean duration of homelessness in days (SD)**	**TBI assessment method**	**Rate of TBI among sample**
Oddy et al. (2012)	12	11 Homeless hostels and day centre services in the UK (dry and wet hostels and day centres)	N=100 (75%); 32.7 (12.3) NR	Self- Report: “Have you ever had an injury to the head which knocked you out or at least left you dazed, confused or disoriented?”	48%
Hux et al. (2009)	11	Homeless shelter in a midwest State (USA)	N=240 (14%); 35.6 (NR); NR	HELPS screening Tool; asks five questions related to events where respondents may have hit their head, the aftermath associated with the event and problems of daily living	20%
Hwang et al. (2008)	15	Shelters and meal programs in Toronto, Ontario (Canada)	N=904 (67%); 37.4 (12.9); 1606 (2154)	Self- Report: “Have you ever had an injury to the head which knocked you out or at least left you dazed, confused or disoriented?”	53%
Kim et al. (2007)	12	Neurosurgical department (Korea)	N = 76 (93%); 40 (53%) were 50 years old or above^2^; NR	Retrospective review of medical records and radiological films following admission to neurosurgical department	NA^3^
Solliday- McRoy et al. (2004)	10	Large homeless shelter in Milwaukee, Wisconsin (USA)	N=90 (100%); 41.0 (9.06); 87.7 (75.6)	Self-Report Questionnaire	48%
Gonzalez et al. (2001)	12	Health Care for the Homeless Clinic in Miami, Florida (USA)	N=60 (60%); 39.8 (11.4); NR	Documented instance of concussion or loss of consciousness or the patient's self-report	38%
Cotman & Sandman (1997)	10	Homeless residents of an 18 month residential program in Orange County, California (USA)	N=24 (54%); 30.6 (6.5); NR	Self-report	8%
Bremner et al. (1996)	15	Hostel in London (UK)	N=62 (100%); NR; median duration of last episode, 42 days^4^	Detailed semi-structured questionnaire to assess health history; Head injury was defined as “sufficient to lose consciousness at some point in their life”	46%

NR, Not reported.

^1^Downs and Black quality checklist score; ratings between 12-17 considered good, 6-12 moderate, and <6 poor.

^2^Mean age not reported, 36 participants were younger than 50 years old, 40 participants were 50 years old or older.

^3^All participants included had a TBI as participants were recruited from a neurosurgical department.

^4^Participants were entrants to a homeless shelter therefore current duration of homelessness was not reported.

### Methodological quality

Using the Downs and Black Checklist [20], the median methodological quality of the studies reviewed was 12 (range 10–15). The most commonly identified limitations were due to lack of a power calculation and poor external validity. Specifically, none of the studies reported a power calculation for main effects, and only one study [21] provided sufficient information to be able to determine that the study sample was representative of the target population.

### Setting/location

In four of the eight studies [21-24] samples of homeless persons were exclusively recruited from residential or shelter settings, meaning no unsheltered persons were included in the sample. In three studies, homeless persons were either recruited from both shelters and meal programs [7], wet and dry hostels and day centres [25], or a health care clinic for homeless people [26]. In one study [27] all homeless participants were neurosurgical patients who were admitted to the emergency room of a Korean hospital due to head trauma. Four of the eight studies were conducted in the USA [22-24,26], two in the UK [21,25] one in Canada [7] and one in Korea [27]. Generally there was limited information to adequately describe the varied research settings, including types of programs offered, the eligibility criteria for shelter or program entrance, and the types of clientele served. In addition, most studies were conducted at single sites/locations within one jurisdiction. No multi-city studies were identified.

### Sample

Across the eight studies, sample sizes were generally small. The median number of homeless persons sampled between all eight studies was 83 (range 24–904). Sample characteristics related to duration of homelessness were not well described. For example, information on duration of homelessness was only reported in three studies [7,21,24]. There were limited samples of females and almost no youth or children studied. Specifically, homeless youth (above the age of 16) were only included in two studies [7,23] and homeless children were only included in one study [23]. Although females were included in six [7,22,23,25-27] of the eight identified studies, in all but one [23], males made up more than half of the study sample. The majority of homeless persons sampled in the identified studies were adult males, who were on average 36.2 years old (range 30.6-41.0). However, two studies were not included in the calculation of average age of homeless participants because age not reported [21] or was reported based on decade [27].

### Outcome measures

The majority of studies relied on self-reported questions to determine a history of TBI. A valid screening tool for TBI, the HELPS screening tool, was only used in one study [23]. The HELPS required respondents to answer five general questions about TBI events and any aftermath that may have been associated with that event. For example, respondents were asked whether they had hit their head or been hit on the head and then further questioned as to whether they were seen in the hospital following the event or had experienced certain problems in daily living following the event. Medical records were reviewed in only two studies [26,27]. There were no studies with repeat assessments and no longitudinal design studies. A control group of people from the general population was used in only two studies [25,27]. The rate of TBI among the homeless persons sampled varied across studies, with reported TBI ranging from 8% to 53% of those sampled. The lowest rate of TBI was reported by Cotman and Sandman (2007), who recruited participants from a setting with the most specific entrance criteria, a residential program that did not admit persons with mental illness or developmental delay.

Injury details, such as severity, and age at time of injury were only explored in three studies [7,25,27] (Table 2). In two studies, the majority of the participants reported that their first [25] or most severe TBI [7] was mild. However, in one study 69% of homeless participants were found to have moderate and severe TBI based on Glasgow Coma Scale score on hospital admission [27]. Hwang and colleagues (2008) found that mean age at first TBI was 19.9 years old while Oddy and colleagues (2012) determined that first age of TBI was 17.8 years old. Although Kim et. al (2007) did not explore age at first injury, the participants with TBI in their sample who were homeless were significantly younger than those who were not homeless. Lifetime number of TBIs, the temporal relationship between onset of TBI and duration of homelessness, and ethnic differences were only reported in two studies [7,25]. Both studies found that 60% of the homeless individuals who reported a history of TBI had experienced more than one TBI, suggesting an increased susceptibility to injury. While Hwang and colleagues found that for 70% of participants with a history of TBI, the first incidence occurred before the onset of homelessness, Oddy and colleagues determined that the occurrence of TBI preceded homelessness for 90% of their sample with a history of injury. Moreover, Hwang and colleagues determined that a history of TBI was more common among white males in their sample, while Oddy and colleagues found no significant differences between the *numbers* of injuries reported by homeless males as compared to homeless females.

**Table 2:** Injury-related details reported

**Research study**	**Injury severity**	**Mean age at first injury**	**Injury preceded homelessness (%)**	**Sex differences**	**Lifetime history of TBI**
Oddy et al. (2012)	81% mild	19.9	90%	TBI more prevalent among males in the sample; no significant sex related difference between number of injuries	52.1% reported more than one injury
Hwang et al. (2008)	66% mild	17.8	70%	TBI significantly more common among white males in the sample	60% reported more than one injury
Kim et al. (2007)	64% moderate or severe	Homeless participants with a TBI significantly younger than non-homeless pariticipants^1^	NR	NR	NR

NR, Not reported.

^1^Age at first injury not documented; at time of admission to the neurosurgery department for TBI, homeless participants were significantly younger than non-homeless participants.

Possible related outcomes or factors associated with TBI among homeless people were only examined in two studies [7,24]. Hwang and colleagues (2008) found that a history of TBI was significantly associated with seizures, mental health and drug problems, and poorer physical and mental health status [7]. Solliday-McRoy and colleagues (2004) examined the relationship between a history of TBI and neuropsychological test scores. However, no significant differences between those with a history of TBI and those without were found and loss of consciousness was not related to any of the neuropsychological test scores [24].

## Discussion

This systematic review of research studies examining the link between TBI and homelessness identified very few articles on the topic. In comparison, a search of the terms “homeless” AND “substance abuse” conducted at the same time yielded almost 2,000 articles in MEDLINE/Pubmed. We are confident that our review has captured all of the information available to date, as we used comprehensive search strategies and contacted authors of published articles to solicit any additional studies. Care was taken to thoroughly review each identified source and extract pre-determined data elements.

The findings of the few studies identified pointed to high rates of TBI among people who are homeless. Moreover, the two studies that explored the temporal relationship between homelessness and TBI [7,25] revealed that for the majority of participants, the first incidence of TBI occurred before the onset of homelessness, suggesting that TBI may be a risk factor for homelessness. Although not widely explored among the research reviewed, it is also possible that another factor, such as impulse control disorders, may predispose individuals to both TBI and homelessness. For example, Hwang and colleagues (2008) found that alcohol and drug problems were significantly more common among those with a TBI in their sample. Similarly, and in line with findings from the general public, the current review also suggests that homeless individuals with a history of TBI have an increased susceptibility to subsequent injuries [7,25] as is illustrated by Oddy et al. and Hwang et al.’s findings that among those with a history of TBI, over 60% had experienced more than one. This is noteworthy as previous literature points to the potential for multiple TBIs to have cumulative adverse impacts on cognitive functioning [28]. Previous literature also demonstrates that cognitive impairment may contribute to the chronicity of homelessness [12]. When reported, injury severity tended to be mild in the homeless samples studied in the shelter and meal program settings [7,25] and moderate to severe in the neurosurgical department setting [27]. However, this finding was only examined in three studies, and in one of the studies severity of injury was only determined for first injury, not for the most severe injury. It is possible that a participant from this study may have had a mild TBI and subsequently incurred more severe injury. Furthermore, it is possible that those with less severe TBIs do not access emergency rooms as frequently as those with more severe injury, explaining why those examined in the neurosurgical department setting may have been found to have more severe TBIs.

The findings of this review point to sex-related differences in the prevalence of TBI among individuals who are homeless as the majority of studies that included mixed sex samples illustrated a greater likelihood of TBI in males who are homeless [7,25]. Although Hux and colleagues (2009) did not explore sex differences among their homeless sample, [23], their sample was predominantly female and yielded one of the lowest prevalence rates of TBI as compared with samples of homeless people from the other studies. It is also important to note that Oddy and colleagues (2012) found no differences between males and females in terms of the number of TBIs acquired. However, they reported a greater prevalence of TBI among males (83%) as compared to females (17%). These findings align with the increased rates of TBI amongst males in the general population [29].

The methodological quality of the studies included in this review, as assessed by the Downs and Black (1998) checklist, was generally moderate. The most commonly identified limitation of the studies was limited external validity and reporting of power calculations. Specifically, information was frequently missing to enable the reader to determine whether the study sample was representative of the entire source population and whether or not the study was sufficiently powered to detect differences in homeless samples with a history of TBI and without a history of TBI. These findings suggest that there is a need for more representative sampling in future studies. This can be achieved through random stratified sampling and demonstrating statistically that included participants are similar in demographics to the target population. Power calculations should also be included in future studies.

### Limitations of the evidence base

The majority of the studies did not primarily aim to address TBI among people who are homeless. As a result, history of TBI was assessed through very basic questions (see Table 1) and the questions were only asked using a validated measurement tool in one of the eight identified studies [23]. Validated screening tools for TBI such as the Ohio State University Traumatic Brain Injury Identification Method **(**OSU TBI-ID) and the Brain Injury Screening Questionnaire (BISQ) should be used in future studies to provide a more reliable estimate of rate of TBI. Ideally, studies could have objective measures of TBI such as advanced neuro-imaging. TBI was not consistently defined across the studies. For example, in some studies [21,24] the determination of injury was dependent on whether an individual had lost consciousness. However, it is generally accepted that loss of consciousness is not necessary for a diagnosis of TBI [9]. Consequently, estimates of the rates of TBI among certain study samples may have been conservative. In future studies TBI should be conceptualized in keeping with standard diagnostic criteria. Studies may also not have been adequately powered to examine questions related to history of TBI. As well, a majority of the studies did not use randomized approaches to recruitment, leaving the possibility of selection biases.

Homeless persons who were not sheltered were only included in four studies [7,25-27]. Hwang and colleagues (2008) published one of the largest studies, which recruited homeless persons from both shelters and meal programs. Oddy and colleagues (2012) recruited from both day centres and hostels, Kim et al. recruited for a neurosurgery department, and Gonzalez and colleagues (2001) recruited from a health care clinic for homeless people. This variation in sampling is noteworthy, as there may be significant differences in terms of physical and mental health between those who access shelters and those who do not [30,31]. Although it may be harder to access homeless persons living on the streets, future studies are necessary in order to examine the occurrence of TBI among unsheltered homeless persons. It may be useful to examine the difference in reported TBI among those who access shelters as compared with homeless individuals who do not in order to determine the risk factors for TBI among people who are homeless. This information may also be valuable in guiding prevention and interventions strategies to the groups of homeless individuals who are most at risk of suffering a TBI.

Few of the reviewed studies provided injury related details such as severity or symptoms related to TBI. None of the studies reported on mechanism of injury. Only three studies provided details on the severity of injury, and age at injury [7,25,27], and only two of these studies reported on the temporal relationship between TBI and onset of homelessness [7,25]. As previously mentioned, gender differences were not widely explored in these studies. There were also no studies that empirically assessed those who provide services to the homeless (e.g. shelter staff, clinicians, nurse practitioners) on their current practices or knowledge related to TBI.

The existing body of literature also lacked qualitative explorations of homelessness and TBI. Providing evidence from qualitative research has been identified as an important means of tailoring intervention programs in order to increase the likelihood for successful uptake [32]. Such research provides personal insight into TBI, emphasizes the importance of persons with TBI receiving empathy and respect, and increases professionals’ understanding of the challenges that survivors face [33]. TBI research should include qualitative methods in order to gain deeper insight into the experience of being homeless with a brain injury. Qualitative data could be used to evaluate and develop sustainable and appropriate interventions.

Outside of peer-reviewed publications, there is also a lack of information on the prevalence of TBI among people who are homeless. A scoping review of grey literature found few sources available online or in print on this topic. For example, the few resources found included an article featured in the Wall Street Journal [34], a four part series in a newspaper sold by individuals who were homeless in Portland, Oregon [35-38], a series of articles available through the National Health Care for the Homeless Network (e.g. [6]) and previous conference presentations that discuss the issue of TBI among homeless persons (e.g. [39]). These limited sources pointed to a clear demand for more information on TBI and homelessness and highlight the lack of empirical data to support educational and awareness initiatives. The few identified sources that provide information on TBI and homelessness were mostly geared towards care providers, clinicians, and academics. Very little information was available for homeless persons themselves or for policy makers who influence funding decisions. In the future, networks such as the Health Care for the Homeless Clinics’ Network that provides many resources on this topic, could be leveraged for knowledge translation. As well, newspaper articles geared towards the general public and to homeless populations are a valuable way to disseminate knowledge.

## Conclusions

Although the rates of TBI among people who are homeless people vary between the studies in this review, all of the results suggest that this population experiences a disproportionately high risk for TBI as compared to the general population [40]. There is clearly a strong need for carefully designed, larger-scale future research studies in this area. Such studies should assess TBI using validated measurement tools, collect adequate descriptive data regarding the research setting and study population, include under-researched samples such as women and youth as well as look at the needs and knowledge level of care providers. Future studies should also employ qualitative as well as quantitative methodologies, and longitudinal designs. Clearly, any study on persons who are homeless needs to include a measure of history of brain injury and relevant sequelae to adequately assess their health issues, which has been notably absent in the literature related to homelessness to date. The results of these studies should also be disseminated to key stakeholders such as person who are homeless, care providers, healthcare professionals, and policy makers in an accessible format. Most importantly, strategies for best addressing the effects of TBI need to be researched to prevent and reduce homelessness among this vulnerable population.

## Abbreviation

TBI: Traumatic brain injury.

## Competing interests

The authors declare that they have no competing interests.

## Authors’ contributions

JTV conceived of the study, participated in the design of the study, title and abstract screening, full text screening, data extraction and analysis and has helped draft the manuscript, NE participated in title and abstract screening, full text screening, data extraction and analysis and drafted the manuscript, AC has been involved with data extraction and interpretation, and has contributed to the draft and critically revising the manuscript for intellectual content, MC has been involved in interpretation of data, drafting and critically revising the manuscript for intellectual content, SH has been involved in study design and interpretation of data, drafting and critically revising the manuscript for intellectual content, PK has been involved in interpretation of data, drafting and critically revising the manuscript for intellectual content, DO has been involved in interpretation of data, drafting and critically revising the manuscript for intellectual content and VS has been involved in interpretation of data, drafting and critically revising the manuscript for intellectual content. All authors read and approved the final manuscript.

## Pre-publication history

The pre-publication history for this paper can be accessed here:

http://www.biomedcentral.com/1471-2458/12/1059/prepub
